# A post-MI power struggle: adaptations in cardiac power occur at the sarcomere level alongside MyBP-C and RLC phosphorylation

**DOI:** 10.1152/ajpheart.00899.2015

**Published:** 2016-05-27

**Authors:** Christopher N. Toepfer, Markus B. Sikkel, Valentina Caorsi, Anupama Vydyanath, Iratxe Torre, O'Neal Copeland, Alexander R. Lyon, Steven B. Marston, Pradeep K. Luther, Kenneth T. Macleod, Timothy G. West, Michael A. Ferenczi

**Affiliations:** ^1^Molecular Medicine Section, National Heart and Lung Institute, Imperial College London, London, United Kingdom;; ^2^Laboratory of Molecular Physiology, National Heart and Lung Institute, National Institutes of Health, Bethesda, Maryland;; ^3^Department of Cardiac Medicine, National Heart and Lung Institute, Imperial College London, London, United Kingdom;; ^4^Laboratoire Physico-Chimie, UMR168, Institute Curie, Paris, France;; ^5^Nationa Institute of Health Research Cardiovascular Biomedical Research Unit, Royal Brompton Hospital, London, United Kingdom;; ^6^Royal Veterinary College London, Structure & Motion Laboratory, North Mymms, United Kingdom; and; ^7^Lee Kong Chian School of Medicine, Nanyang Technological University, Singapore

**Keywords:** contractile function, infarction, regulatory light chain, myosin binding protein-C, contractile apparatus

## Abstract

Myocardial remodeling in response to chronic myocardial infarction (CMI) progresses through two phases, hypertrophic “compensation” and congestive “decompensation.” Nothing is known about the ability of uninfarcted myocardium to produce force, velocity, and power during these clinical phases, even though adaptation in these regions likely drives progression of compensation. We hypothesized that enhanced cross-bridge-level contractility underlies mechanical compensation and is controlled in part by changes in the phosphorylation states of myosin regulatory proteins. We induced CMI in rats by left anterior descending coronary artery ligation. We then measured mechanical performance in permeabilized ventricular trabecula taken distant from the infarct zone and assayed myosin regulatory protein phosphorylation in each individual trabecula. During full activation, the compensated myocardium produced twice as much power and 31% greater isometric force compared with noninfarcted controls. Isometric force during submaximal activations was raised >2.4-fold, while power was 2-fold greater. Electron and confocal microscopy demonstrated that these mechanical changes were not a result of increased density of contractile protein and therefore not an effect of tissue hypertrophy. Hence, sarcomere-level contractile adaptations are key determinants of enhanced trabecular mechanics and of the overall cardiac compensatory response. Phosphorylation of myosin regulatory light chain (RLC) increased and remained elevated post-MI, while phosphorylation of myosin binding protein-C (MyBP-C) was initially depressed but then increased as the hearts became decompensated. These sensitivities to CMI are in accordance with phosphorylation-dependent regulatory roles for RLC and MyBP-C in crossbridge function and with compensatory adaptation in force and power that we observed in post-CMI trabeculae.

## Abstract (precis)

*Compensation postchronic myocardial infarction (CMI) in rats is characterized in trabeculae as increased force and power production during physiological shortening, which occurs alongside classical hypertrophy. Sarcomeric contractile gain is influenced by mechanisms involving reduced myosin binding protein C (MyBP-C) and raised regulatory light chain (RLC) phosphorylation*.

## NEW & NOTEWORTHY

*Compensation postchronic myocardial infarction (CMI) in rats is characterized in trabeculae as increased force and power production during physiological shortening, which occurs alongside classical hypertrophy. Sarcomeric contractile gain is influenced by mechanisms involving reduced myosin binding protein C (MyBP-C) and raised regulatory light chain (RLC) phosphorylation*.

chronic myocardial infarction (CMI) is typified by the obstruction of blood flow in the coronary arteries, which creates cardiac remodeling in surrounding tissue regions not directly affected by the infarct (25, 66). Cardiac remodeling follows a time course, which displays Initial compensatory hypertrophy (myocardial compensation), which typically deteriorates into systolic and diastolic dysfunction (myocardial decompensation): heart failure (HF) (54). Alongside cardiac remodeling, many posttranslational modifications (PTMs) have been found to occur (1).

Cardiac power, shortening velocity, and force are fine-tuned by direct sarcomeric protein phosphorylation (58). For example, a 40% increase in regulatory light chain (RLC) phosphorylation doubles power production in healthy cardiac muscle, suggesting that phosphorylation of RLC, and possibly of other sarcomeric proteins, is a key adaptive response of the myocardium to CMI (58). Our previous findings indicate that RLC phosphorylation is indeed affected by CMI (58). What has not been clear in the literature is the effects of CMI on the mechanics of tissue distant from the infarct zone and what these PTMs might be doing to cardiac mechanics. Previous work has either identified structural changes to the myocardium in the border zone (67) or changes in isometric function with acute occlusion (32). There is a paucity of data relating measurements of in vivo organ function, ex vivo muscle mechanics, and tissue structure (33, 42, 44), specifically relating to both remodeling phases. Additionally, previous studies have predominantly interrogated isometric force with pCa-tension measurements and not force, velocity, and power measurements during physiological shortening (10, 11, 32, 68). There is evidence to suggest that isometric force production is largely unaffected by heart failure, prompting our investigations to understand these effects at the sarcomeric level using power output as our determinant of contractile change (18).

Here we test the hypotheses that *1*) permeabilized cardiac trabeculae, extracted from sites distant from the infarct zone, are “compensated” (e.g., displaying greater contractile power and increased RLC phosphorylation) in a CMI model, which is not accounted for by the hypertrophic response (34); and *2*) that contractile characteristics parallel RLC phosphorylation level enrichment in disease progression. We measure the phosphorylation levels of myosin binding protein C (MyBP-C) and troponin I (TnI), which influence muscle mechanics, to test the hypothesis that sarcomeric protein alteration is a phenomenon of sarcomeric level contractile compensation (9, 16, 29, 51, 63).

Force-velocity (FV) measurements provide a robust methodology for exploring changes in cross-bridge mechanics during physiological shortening (64) in permeabilized trabeculae, in a disease model. From FV measurements we can determine whether peak power, velocity, force at peak power, and peak unloaded shortening velocity (*V*_max_) are altered by compensation and decompensation. We used full activation (32 μM [Ca^2+^]), and partial activation (1 μM [Ca^2+^]) to ascertain whether the mechanics are altered by MI in a consistent manner, independently of [Ca^2+^]. In addition we interrogated changes in the morphological features of the exact same trabecula preparations using electron microscopy (EM) and confocal microscopy to assess type I collagen abundance. Confocal two-photon excitation (TPE) and s harmonic generation (SHG) microscopy was used to assess relative collagen abundance and infiltration (4). The process of collagen deposition and infiltration has been classified as reparative and reactive to necrosis and is often linked with chronic heart failure (3, 55). EM was used to validate these observations at higher resolution.

This study couples mechanical-morphological observations with in-vivo clinical measures of heart function. Bringing these data together highlights that contractile protein phosphorylation is important in the compensatory mechanism of increased mechanical output, defined by physiological shortening under load. We hypothesize that posttranslational modification alters muscle function at the cross-bridge level and propose how sarcomeric protein alterations affect trabecular force, shortening, and power in this disease.

## METHODS

### 

#### Chronic myocardial infarction model generation.

All animal surgical procedures and perioperative management were carried out in accordance with the *Guide for the Care and Use of Laboratory Animals* published by the U.S. National Institutes of Health under assurance number A5634-01 and also conformed to the UK Animals (Scientific Procedures) Act 1986. Imperial College Ethical Review Committee and the Project License authorized these studies in accordance with the United Kingdom Home Office Animals (Scientific Procedures) Act 1986. Adult male Sprague-Dawley rats (250–300 g) (*n* = 18) underwent proximal left coronary artery ligation to induce myocardial infarction (MI) as described previously (34). Sixteen unoperated rats were used as age-matched controls (62).

#### Trabecular preparation and experimental procedure.

Explanted rat hearts (Sprague-Dawley) were immediately rinsed with oxygenated ice-cold Krebs-Henseleit solution. Cylindrical trabecular preparations (50–200 μm in diameter and 0.8–2 mm in length) were excised from the inner wall of the left ventricle superior to the left ventricular papillary muscle distant from the anterior apical infarct region. T-clips were crimped onto the ends of the trabeculae and the tissue was permeabilized by immersion in a 2% Triton solution. The samples were attached to the experimental rig by hooks and shellac was applied to the ends of the trabecular segments to minimize compliance of the damaged ends of the preparations. Sarcomere length was set to 2.1 μm by laser light diffraction assessed both before and after activation cycles. Preparations were activated and held at isometric tension for 1 s to make sure there was no slippage of the preparation. Tension was measured at 20°C in an activating solution with either 1 or 32 μmol/l free [Ca^2+^], corresponding to physiological, or maximal activation, respectively. FV and slack test experiments were carried out as previously described (58) using release-ramp maneuvers to obtain stable plateaued forces during isotonic shortening at set velocities.

#### FV relationship.

Force and power measurements were normalized per cross-sectional area (CSA) and tissue volume (mm^3^) respectively, with peak unloaded shortening velocity (*V*_max_) reported in muscle lengths per second (ML/s). Each FV relationship was fit to a hyperbola: *V* = *a* × *V*_max_ × (1 − P/P_o_)/(P + *a*) described by Hill (1938) (20). The *V*_max_ measurements were obtained by the slack-test method (14) and constrained the FV fitting procedure. The fitting parameters are presented as means ± SE and the corresponding n values for each dataset is stated.

#### TPE and SHG microscopy.

TPE and SHG microscopy measurements were performed to measure the abundance of fibrosis in trabecular samples. After the FV relationship was determined in each trabecula, the very same trabecula was mounted on a custom-made microscope stage. A Leica TCS SP5 upright laser scanning system (Leica Microsystem), coupled to a titanium:sapphire laser (Spectraphysics Mai Tai 690–1020 nm, 90 MHz; Spectra-Physics, Santa Clara, CA), was used. Multiphoton imaging was performed at 900 nm to maximize backward SHG collection (440–460 nm) and autofluorescence detection (500–700 nm) by using a water immersion objective (×25 water 0.9 NA, Zeiss). Optical sections were simultaneously acquired collecting both autofluorescence and SHG for a volume depth of 150–200 μm (z step size: 0.4 μm). Intensity based and volumetric analyses were performed to calculate the change in fibrosis in MI samples compared with age-matched controls (AMCs; as previously described in Ref. 4).

#### Electron microscopy.

Hearts were excised and arrested in preoxygenated Krebs solution and immediately fixed using 3% glutaraldehyde. Trabeculae were dissected from the LV and fixed in 1% osmium tetroxide. Muscles were dehydrated by incubation in acetone and embedded into epoxy resin Araldite CY 212 (Agar). Ultra-thin (80 nm) sections were cut, picked on copper grids, and stained with 2% uranyl acetate and Reynolds lead citrate. All electron micrographs were acquired at 100 kV using a JEOL 1200 EX electron microscope operated at 100 kV and recorded directly onto a CCD camera (Tietz Fastscan F114). Montages of 1,000-μm^2^ (30 × 34 μm^2^) longitudinal sections were performed by acquiring 12 sequential electron micrographs of representative tissue areas at a magnification of ×1,000. Separate micrographs were stitched together using Fiji processing package (ImageJ).

#### Phos-tag analysis of protein phosphorylation.

The phosphorylation status of TnI, MyBP-C, and RLC was assessed in controls and during the progression of disease post-MI. The techniques used for quantification of the gel assay have been described previously (8, 36, 58).

#### Statistics.

One-way ANOVA was used to identify differences between the clinical parameters of the model and phosphorylation levels. Significance was assigned to values that reached *P* < 0.05 after the use of Tukey's correction. Two-way ANOVA was used to compare the contractile parameters of the experimental treatment groups. A significance cut-off of *P* < 0.05 was used for all analysis.

## RESULTS

### 

#### From compensation to decompensation: myocardial hypertrophy and in vivo function of the CMI model.

**Fig. 1.**
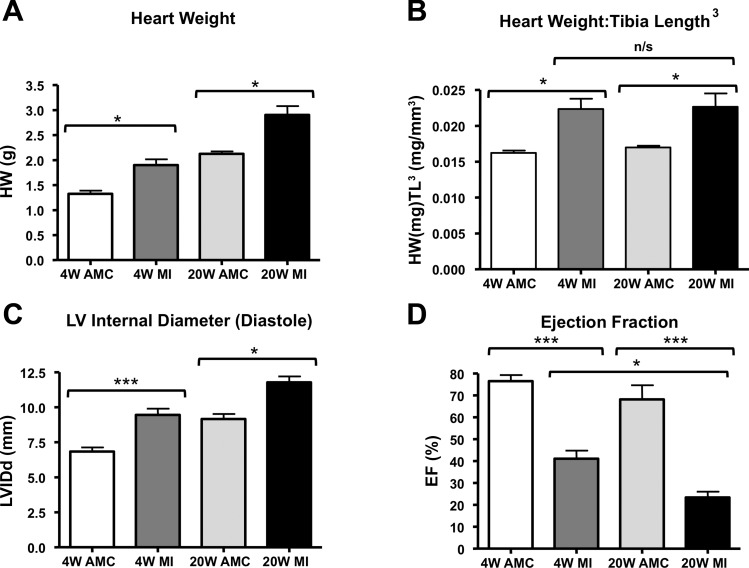
Biometric and echocardiographic measures of cardiac hypertrophy. *A*: heart weight measurements in grams. *B*: heart weight per tibia length^3^ (HW:Tibia Length^3^) measuring the degree of hypertrophy at both time points. *C*: measurement of left ventricular (LV) internal diameter indicating LV dilatation at both MI4W and MI20W. *D*: ejection fraction (EF) shows decline in MI4W and MI20W. (*n* = 4–6 rats in each group, *P < 0.05, ***P < 0.001 by one-way ANOVA with Tukey's correction).

Heart weight was significantly increased among MI animals compared with corresponding unoperated AMCs, indicating developed hypertrophy (Fig. 1*A*). Normalized heart weight defined the degree of hypertrophy between MI4W and MI20W groups, which indicated a similar degree of hypertrophy in the MI4W and MI20W animals (Fig. 1*B*).

Echocardiography showed an increase in LV internal diameter in diastole (LVIDd) of the MI groups (Fig. 1*C*), indicating pathological LV dilatation in MI4W and MI20W cohorts. Ejection fraction (EF) was also significantly reduced in MI groups. EF decreased with age, indicated by the comparison between MI4W and MI20W (Fig. 1*D*).

In vivo measures of heart weight and function confirmed a “compensatory” phenotype in MI4W, and phenotypic “decompensation” at MI20W, which will be denoted as such throughout (34).

#### Contractile gain during compensation.

**Fig. 2.**
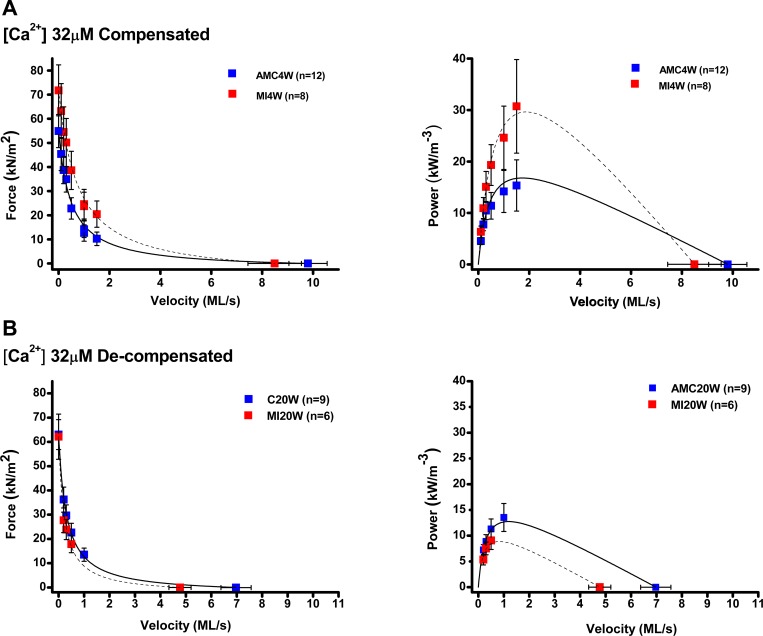
Force-velocity (FV) and power velocity (PV) relations from myocardial infarction (MI)4W, MI20W, age-matched control (AMC)4W and AMC20W trabeculae at 32 μM [Ca^2+^]. *A*: FV and PV relationships from MI4W, red squares, *n* = 8, and AMC4W, blue squares, *n* = 12, at saturating [Ca^2+^] (32 μM). Solid and dotted lines represent the Hill equation fits of trabeculae from control and infarcted animals respectively. *B*: FV and PV relationships from MI20W, red squares, *n* = 6, and AMC20W, blue squares, *n* = 9, at physiological [Ca^2+^] (32 μM).

The features of FV and power-velocity relationships in maximally (32 μmol/l Ca^2+^) and submaximally activated (1 μmol/l Ca^2+^) trabeculae from MI4W and AMC4W are displayed in Fig. 2, *A* and *B*, and tabulated in 2*C*. The degree of activation ([Ca^2+^]_free_), affected the contractile properties. Isometric force, *V*_max_, and peak power were lower in the submaximally activated trabeculae, from both MI4W, and AMC4W hearts. Curvature of the FV relationships (*a*/P_0_) was reduced in submaximal activations, and this effect was accompanied by overall reduced shortening velocity at which peak power is observed (*V*_pp_). Key signs that MI4W hearts were mechanically “compensated” were *1*) isometric force was increased significantly (by 32% in maximal activations and by 140% in submaximal activations, compared with AMC4W; *P* = 0.002); and *2*) peak power was elevated significantly (by 86–88% in both maximally and submaximally activated MI4W trabeculae, *P* < 0.05); and *3*) force at peak power (F_PP_) was raised by MI in both maximal and submaximal activation (82 and 44%, respectively, *P* < 0.05)

Interestingly, there was a significant interaction effect between MI and Ca level on average *V*_max_ (*P* = 0.015), which was manifest as the relatively higher (2-fold) *V*_max_ in the compensated MI4W compared with control AMC4W, at submaximal activation (see Table 1). The average *a*/P_o_ showed a similar interaction effect (*P* = 0.011), where the curvature in the MI4W submaximal activations (0.32 ± 0.19, *n* = 6) was found to be intermediate to the mean values at full activation (ranging 0.05 to 0.13) and the value for AMC4W at submaximal activation (0.98 ± 0.17, *n* = 9). *V*_pp_ was 30% higher in MI4W compared with AMC4W, but the difference was not significant.

**Table 1.** Mechanical parameters from trabeculae at MI4W and AMC4W

	AMC4W at 32 μM (*n* = 12)	MI4W at 32 μM (*n* = 8)	AMC4W at 1 μM (*n* = 9)	MI4W at 1 μM (*n* = 6)
F_0_, kN/m^2^	55 ± 7	72 ± 11†	21 ± 4*	51 ± 8*†
*V*_max_, ML/s	9.8 ± 0.8	8.5 ± 1.0	2.4 ± 0.3*	5.0 ± 0.6*‡
*V*_PP_, ML/s	1.7 ± 0.2	1.9 ± 0.3	1.0 ± 0.1*	1.3 ± 0.1*
PP, kW/m^3^	18 ± 4.0	33 ± 7.9†	8.7 ± 1.7*	16 ± 2.3*†
*a*/P_0_	0.05 ± 0.01	0.13 ± 0.03†	1.00 ± 0.30*	0.32 ± 0.19*†‡
F_PP_, kN/m^2^	9.6 ± 1.5	18 ± 4.5†	8.9 ± 1.4	13 ± 2.2†
F_PP_/F_0_	0.17 ± 0.01	0.24 ± 0.06†	0.43 ± 0.02*‡	0.25 ± 0.01*†
CSA, μm^2^	9,161 ± 1,744	6,608 ± 1,686	4,416 ± 530	4,732 ± 871

Values are means ± SE. Mechanical parameters for myocardial infarction and age-matched controls at 4 wk (MI4W and ACM4W) trabeculae are tabulated. F_0_, peak isometric force; *V*_max_, peak unloaded shortening velocity; *V*_PP_, velocity at which peak power is attained, PP is the peak power; *a*/P_o_, curvature of the hyperbolic fit of the force-velocity data; F_PP_ is the force at peak power; F_PP_/P_o_; ratio of force at peak isometric force; CSA, cross-sectional area of the trabeculae; ML, muscle length.

* *P* < 0.05, significant [Ca^2^^+^] effect.

† *P* < 0.05, significant effect of myocardial infarction (MI).

‡ *P* < 0.05, interaction effect between [Ca^2^^+^] and MI.

#### Contractile loss during decompensation.

**Fig. 3.**
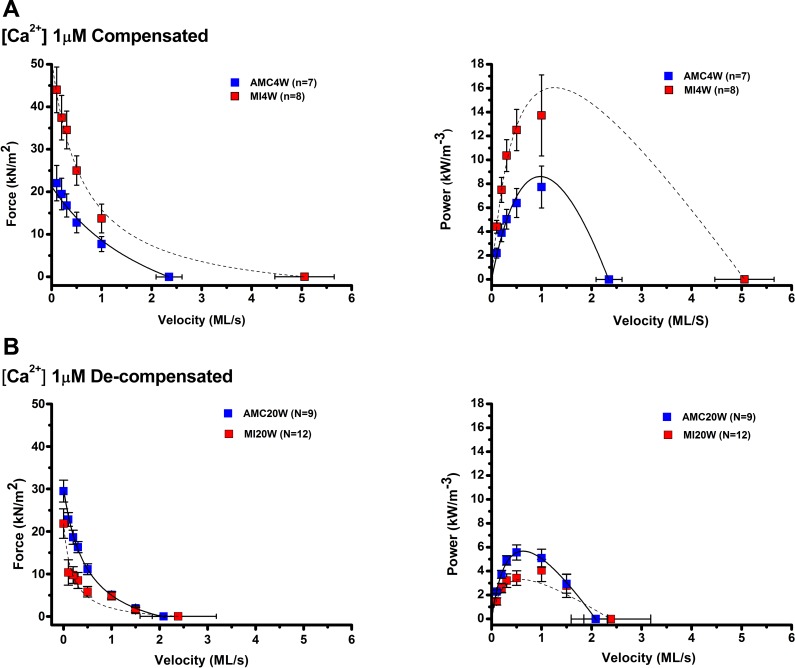
Force-velocity (FV) and power velocity (PV) relations from MI4W, MI20W, AMC4W, and AMC20W trabeculae at 1 μM [Ca^2+^]. *A*: FV and PV relationships MI4W, red squares, *n* = 8 and AMC4W, blue squares, *n* = 7 at maximally activated [Ca^2+^] (1 μM). Solid and dotted lines represent the Hill equation fits of trabeculae from control and infarcted animals respectively. *B*: FV and PV relationships from MI20W, red squares, *n* = 6, and AMC20W, blue squares, *n* = 9 at physiological [Ca^2+^] (1 μM).

At MI20W, most mechanical adaptions observed in the MI4W cohort were reversed (Fig. 3; Table 2). In both fully and partially activated trabeculae, mean isometric forces were not altered by CMI, whereas the same comparisons at MI4W showed that the compensated hearts produced significantly greater isometric force, by more than twofold in partially activated conditions (1 μM [Ca^2+^]_free_). Similarly, the approximate twofold higher average power and *V*_max_ seen in the partially activated compensated hearts, when compared with controls, had reversed in trabeculae from MI20W hearts. In the fully activated decompensated hearts, *V*_max_ was reduced by one-third, while power was 53% of the mean of AMC20W. The main effect of CMI on *V*_max_ and power was observed at full activation, where the control values remained relatively similar to the full activation values for AMC4W. F_PP_ was reduced at MI20W by 20 and 30% in maximal and submaximal activation compared with AMC20W (*P* < 005). Mechanical decompensation was even evident in the fully activated MI20W group. There was a significant interaction effect between MI and Ca level on *V*_max_ and *V*_pp_; both of these mean velocity measures were relatively low in the fully activated MI20W group, compared with the respective AMC20W values.

**Table 2.** Mechanical parameters from trabeculae at MI20W and AMC20W

	AMC20W at 32 μM (*n* = 12)	MI20W at 32 μM (*n* = 8)	AMC20W at 1 μM (*n* = 9)	MI20W at 1 μM (*n* = 8)
F_0_, kN/m^2^	66 ± 6	62 ± 9	30 ± 3*	22 ± 3*
*V*_max_, ML/s	7.2 ± 0.6	4.8 ± 0.4†‡	2.1 ± 0.2*	2.4 ± 0.8*†
*V*_PP_, ML/s	1.2 ± 0.1	0.8 ± 0.1‡	0.7 ± 0.1*	0.7 ± 0.2*
PP, kW/m^3^	14 ± 6.8	7.5 ± 1.4†	5.9 ± 0.5*	4.2 ± 0.6*†
*a*/P_0_	0.05 ± 0.01	0.05 ± 0.08	0.39 ± 0.11*	0.32 ± 0.11*
F_PP_, kN/m^2^	12 ± 1.4	9.5 ± 1.4†	9.4 ± 0.8*	6.3 ± 1.0*†
F_PP_/F_0_	0.18 ± 0.01	0.16 ± 0.01	0.32 ± 0.01*	0.29 ± 0.02*
CSA, μm^2^	12600 ± 2250	10700 ± 2380	20600 ± 2590	32000 ± 4710

Values are means ± SE. Mechanical parameters for MI20W and ACM20W trabeculae are tabulated. F_0_, peak isometric force; *V*_max_, peak unloaded shortening velocity; *V*_PP_, velocity at which peak power is attained, PP is the peak power; *a*/P_o_, curvature of the hyperbolic fit of the force-velocity data; F_PP_ is the force at peak power; F_PP_/P_o_; ratio of force at peak isometric force; CSA, cross-sectional area of the trabeculae; ML, muscle length.

* *P* < 0.05, significant [Ca^2^^+^] effect.

† *P* < 0.05, significant effect of myocardial infarction (MI).

‡ *P* < 0.05, interaction effect between [Ca^2^^+^] and MI.

#### Morphological alterations during disease progression.

**Fig. 4.**
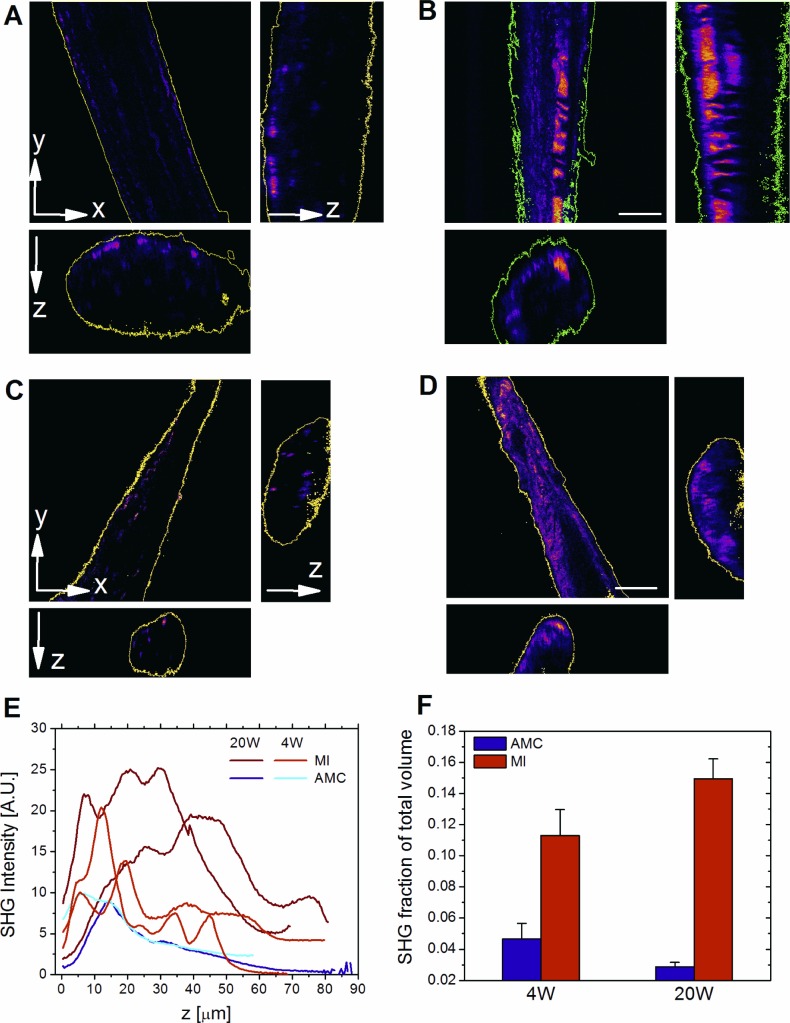
Collagen increase evaluated by two-photon excitation (TPE) and second harmonic generation (SHG) microscopy. 3-Dimensional SHG intensity (shown as *xy*-view and orthogonal views *xz*, *yz*) of one AMC4W sample (*A*) compared with one MI4W sample (*B*), and one AMC20W sample (*C*), compared with one MI20W sample (*D*). Colors ranging from purple to pink denote an increasing intensity of SHG signal. Scale = 25 μm. *E*: SHG intensity; AU, arbitrary units. *F*: SHG fraction of total volume.

TPE and SHG microscopy were used to measure collagen infiltration in the trabeculae used for mechanical experiments. Trabeculae were mounted on a custom-made device for three-dimensional simultaneous recording of SHG signal (at sarcomere length of 2.1 μm), as a collagen marker, and autofluorescence for tissue volume evaluation (Fig. 4) (4). Quantitative volume analysis showed MI4W collagen occupied 2.5-fold more of the tissue volume than in AMC4W and increased to threefold at MI20W (Fig. 4, *B* and *D*). In both cases collagen had become more central in the trabeculae disrupting the myocardial network (Fig. 4, *B* and *D*).

**Fig. 5.**
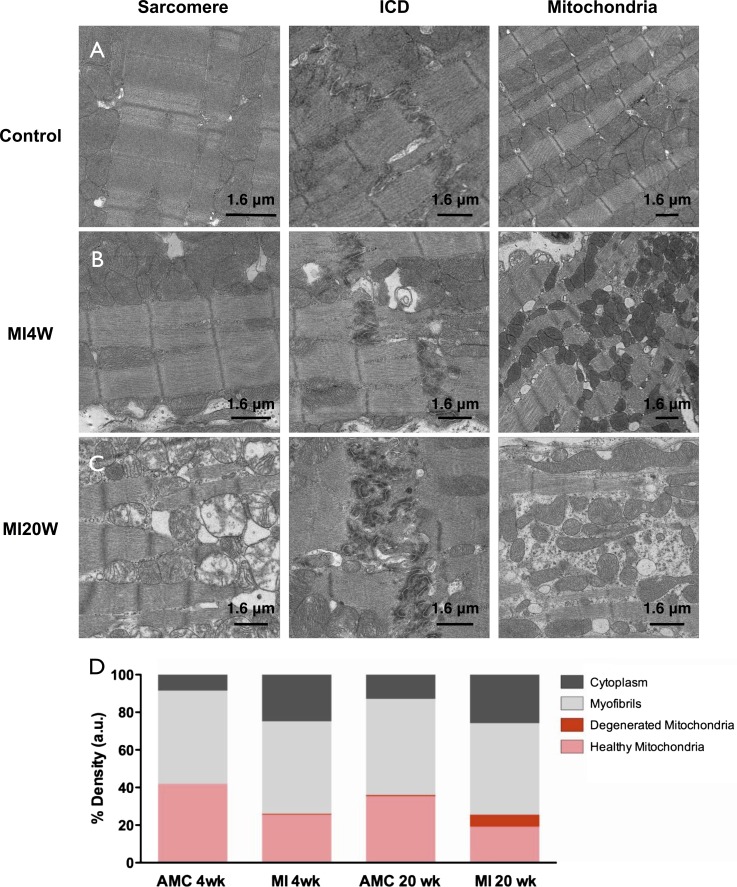
Electron micrographs of left ventricular myocardium. Electron micrographs of age-matched control representative of both control ages (*A*), MI4W (*B*), and MI20W (*C*). Columns show typical micrographs of sarcomere order, intercalated disc (ICD) organization and mitochondrial morphology (Mitochondria), of each treatment group. *D*: quantification of the volume density occupied by mitochondria, myofibrils, and cytoplasm. Myofibrillar contractile volume (MCV) is stated as a percentage in the bar graph.

Myocardial tissue sections, also taken from noninfarct regions of the hearts, were imaged in the electron microscope to confirm the effect of MI on sarcomeric content, structure, and tissue morphology at higher resolution. Control trabeculae, had well ordered sarcomeres, intercalated discs (ICDs) and normal mitochondria (Fig. 5*A*). Age did not affect these structures. At MI4W, tissue disorder was observed in regions not directly affected by CMI, with apparent fibrosis and disordered, shrunken, and rounded mitochondria, ICDs were unaffected (Fig. 5*B*). Changes in mitochondrial morphology have been reported previously for a variety of cardiac conditions (26, 52) and are usually linked to impaired metabolism (2). In MI20W myocardium there was clear evidence of tissue fibrosis with extensive regions of disrupted sarcomeres, highly convoluted ICDs and elongated mitochondria (Fig. 5*C*), which is interpreted as the consequence of organelle fusion events to protect against further hypoxic or ischemic insult (43, 56, 59). Stereological analysis showed that in control tissue the volume of myofibrils was approximately equal to that of normal mitochondria (Fig. 5*D*). During compensation mitochondrial volume decreased and a slight reduction in myofibrillar contractile volume (MCV) was also evident (Fig. 5*D*). There was no increase in the proportion of contractile material in the compensated myocardium.

#### Sarcomeric protein phosphorylation.

**Fig. 6.**
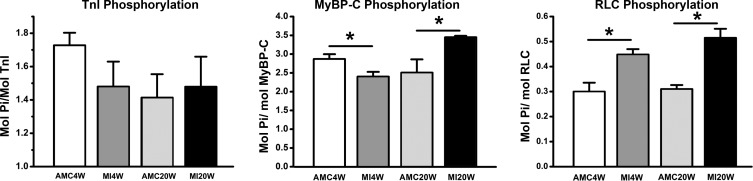
Phos-tag analysis troponin I (TnI), myosin binding protein C (MyBP-C), and raised regulatory light chain (RLC) phosphorylation. All values are denoted as mol P_i_/mol protein. *P < 0.01, phosphorylation differences between infarcted tissues and their corresponding age-matched controls where.

Adaptation during disease progression SDS-PAGE incorporating Phos-tag was used to quantify protein phosphorylation of tTnI, MyBP-C, andRLC (Fig. 6) (8, 35, 58). TnI phosphorylation was unaffected by CMI (Fig. 6). At MI4W MyBP-C phosphorylation was reduced to 2.4 ± 0.1 mol P_i_/mol MyBP-C (*n* = 5) vs. AMC4W 2.9 ± 0.1 mol P_i_/mol MyBP-C (*n* = 5), a 17% reduction (*P* < 0.05) (Fig. 6). Phosphorylation increased by 40% (*P* < 0.05) at MI20W to 3.5 ± 0.4 mol P_i_/mol MyBP-C (*n* = 5) from 2.5 ± 0.4 mol P_i_/mol MyBP-C (*n* = 5) in AMC20W, and RLC phosphorylation increased at MI4W to 0.45 ± 0.02 mol P_i_/mol RLC (*n* = 5) from 0.30 ± 0.04 mol P_i_/mol RLC (*n* = 5) in AMC4W (*P* < 0.05). This was a 50% increase in RLC phosphorylation that was maintained at MI20W 0.52 ± 0.04 mol P_i_/mol RLC (*n* = 5) compared with control at 0.31 ± 0.02 mol P_i_/mol RLC (*n* = 5; *P* < 0.05).

## DISCUSSION

Enhanced power output, isometric force, and force at peak power were consistent features of cardiac compensation in this CMI model. However, hypertrophy and activation level seemed to be only relatively minor determinants of power and force. Cardiac hypertrophy largely reflected the increased collagen deposition in the noninfarcted area of a compensated heart. Mechanical compensation was not observed at the level of the intact organ, likely due to tissue damage in the infarct zone. This highlights the impact of the mechanical compliance and the morphological effect of the scar tissue created by CMI (45). In the present study, hypertrophy in the zone distant from the infarct region was not accompanied by any change in the volume density of the contractile lattice. Hence, factors other than hypertrophy explain a change in power and force per CSA in isolated permeabilized trabeculae. In the present CMI model, calcium availability (1 or 32 μM) had no effect on the MI-dependent relative increases in compensated power and force. It is likely that a key to CMI adaptation lies in changes in sarcomeric protein phosphorylation states, which in turn influence cross-bridge binding kinetics. For example, the increased RLC phosphorylation post-CMI paralleled the upregulation of power and force in trabeculae from compensated hearts, and this change was completely consistent with our previous observations of the effects of exchange of hyper- and hypophosphorylated RLC into trabeculae from noninfarcted hearts (58). Greater RLC phosphorylation in decompensated hearts, while perhaps similarly adaptive at the cross-bridge level, nevertheless coincided with the severe cellular-, organelle-, and sarcomere-level disruptions, all of which likely marked the onset of irreversible cardiac failure in this CMI model.

### 

#### Trabecular mechanics post-MI during compensation.

The contractile apparatus of the MI4W myocardium outperformed AMC4W at both physiological (1 μM) and maximally activating (32 μM) calcium, in spite of the extensive damage in the infarcted region and loss of functional tissue (Figs. 2*A* and 5*B*). These are novel findings in the CMI myocardium, which point to mechanisms of sarcomeric contractile compensation, distinct from a role for tissue hypertrophy. The heightened contractile output of MI4W observed in saturating calcium indicates that the increased performance was not wholly accounted for by changes in calcium sensitivity, which have been well documented previously (12, 61, 68). Therefore, compensation is not completely governed by thin filament regulation. The behavior of the thick filament likely explains calcium-independent compensation, which is a new paradigm for sarcomeric contractile compensation post-CMI. Contractile compensation was not explained by hypertrophy (Figs. 1, *A* and *B*, and 4). Therefore, the existing sarcomeric material had improved its contractile performance (force per CSA and power per volume). Interestingly, the greater influence on power output in compensation was the ability to produce force, not velocity, signified by significant changes in F_PP_ and not *V*_PP_. Therefore, the ability of each contractile unit to produce force during shortening has been fine-tuned post-CMI, independently of hypertrophy.

A straightforward two-state model describes how force production may be altered in muscle (21). This model does not adequately describe force production in muscle, particularly the responses to rapid changes in force and length, but is a useful tool for comparing key parameters in control muscle and during compensation (22, 46). The model has four parameters that can influence force production in muscle. These are: the number of cross-bridges available for cycling (*N*); the force produced by each individual cross-bridge (F); and the fraction of attached cross-bridges (*f*/*f* + *g*), where *f* is the rate constant of cross-bridge attachment and *g* is the rate constant of detachment. Alterations in both the kinetics and mechanics of myosin have previously been observed by altering RLC and MyBP-C phosphorylation.

At MI4W, RLC phosphorylation increased ∼50% in agreement with the findings of others (1, 53). RLC phosphorylation increases calcium sensitivity, force, unloaded shortening, and power produced by cardiac trabeculae (7, 38, 58). RLC phosphorylation increase could either signify a lack of phosphatase (PP1) activity in the myocardium (41), an increase in kinase activity of the two specific kinases, or a mixture of both (5, 6, 13). This phenomenon warrants further investigation. RLC phosphorylation is thought to accelerate *f*, by moving myosin heads closer to actin and thereby increasing the probability of cross-bridge formation (7, 31, 60). By raising *f*, the proportion of cross bridges in load bearing states changes (assuming *g* has not been accelerated to a greater degree) leading to increased isometric force, which we observe at MI4W. Power is also affected at MI4W. Power can be increased either by an increase in force, in velocity, or by both simultaneously. RLC phosphorylation has been previously found to alter *V*_max_ (58). However, in compensation increased power seems to be associated with force rather than velocity. Changes in *V*_PP_ and *V*_max_ are seen in compensation, which are likely a result of acceleration of detachment kinetics, *g* (15). Notably, RLC phosphorylation correlates with changes to both cross-bridge detachment and attachment and with compensation at MI4W. It must be noted that there is also evidence from in vitro motility assays that RLC phosphorylation alters F, the specific force of individual cross bridges, but this has not been addressed in muscle fiber or fibril models (28).

We show here that in MI4W MyBP-C phosphorylation was reduced by ∼17%. Unphosphorylated MyBP-C binds thin filaments and stabilizes thin filament activation (27), while slowing filament sliding (47, 49, 50, 65). Reduced MyBP-C phosphorylation correlates with increased sarcomeric mechanical output in low calcium conditions (Fig. 2*B*). Reductions in MyBP-C phosphorylation move myosin heads away from actin (7). This appears to counteract the effects of RLC phosphorylation. In submaximal calcium, enriched RLC phosphorylation increases cross-bridge proximity to actin. Reduced MyBP-C phosphorylation may actually facilitate cross-bridge attachment in these conditions by augmenting thin filament activation, therefore increasing actin binding site availability for the myosin heads (primed due to the RLC phosphorylation level increase) and actual binding (27). However, this behavior comes at a cost of reduced shortening velocity (47, 49, 50, 65), which is in balance with the accelerated myosin kinetics created by RLC phosphorylation (15, 58). More work is needed to determine whether such tug of war between reduced MyBP-C and increased RLC phosphorylation explains the MI4W mechanical phenotype we see in this CMI model.

It is interesting to note that TnI phosphorylation was not altered by MI, inferring that effects at physiological calcium concentration arise because of increased cooperative activation and not by increased thin filament activation (reviewed Ref. 30). In submaximal calcium, the effects of thin filament activation, in the MI phenotype, seem to outweigh effects due to reductions in sarcomere shortening velocity.

The observed changes in sarcomeric protein phosphorylation provide further evidence that mechanical compensation is the mechanism that accounts for increased force and power in the myocardium post-CMI, which is independent of tissue hypertrophy. Further investigations should be made to directly quantify other sarcomeric protein phosphorylation levels, MHC compositions (24, 37, 39), and titin compositions to identify other possible modulators of muscle mechanics (48). These are important further investigations as murine heart failure has shown a progressive shift from α- to β-myosin heavy chain (β-MHC) expression, which likely occurs in our model (10). Fibers containing β-MHC shorten more slowly and produce less power over a range of applied loads. Therefore, any such α- to β-MHC shift does account for increased power at MI4W (19) but correlates well with mechanical decompensation observed at MI20W. Changes of titin isoform expression are also known to occur in ischemic heart disease in both humans and rats (40). Titin isoform composition was shifted from stiffer N2B to the more compliant N2BA isoform, in a left anterior descending coronary artery occlusion model in rats (40). A loss in myocardial stiffness could lead to a reduced Frank-Starling effect (57), which may need to be compensated for by increased myocardial contractility, as we describe above.

#### Trabecular mechanics post-MI during decompensation.

Mechanical gain was reversed in decompensated myocardium typified by dramatic tissue disorder (Fig. 5*C*) (17). Evidently the loss in power at MI20W is related to reductions in F_PP_, as *V*_PP_ is unchanged. Collagen volume was unaltered; however, it had become more centrally located, which might partially account for reductions in force (Fig. 4, *B*–*D*). This accompanied severe disruption of the sarcomeric environment, ICDs, and mitochondrial structure (Fig. 5*C*). Contrasting the contractile performance of isolated myofibrils, from decompensated hearts with that of compensated, or controls, defines the role played by tissue reorganization in disease progression, as opposed to the direct effect of sarcomeric protein modifications. Indeed, an increased volume of abnormal mitochondria (40% of total mitochondrial volume) as observed in Fig. 5*D* correlates with reduced force production. Clearly altered energy production in the tissue could be driving decompensation and warrants further investigation in the context of this model. During decompensation there is evidence of high cytosolic calcium, due to loss of calcium homeostasis; therefore, phosphorylation of MyBP-C may be triggered by chronic high cytosolic calcium, reducing its binding to actin, which reduces thin filament activation (27). Theoretically, this could be an adaptation that counteracts diastolic insufficiencies created by altered relaxation kinetics. However, at this late time point in disease it becomes difficult to appraise muscle mechanics, due to the overbearing tissue disorder.

#### Concluding remarks.

We have observed novel mechanical adaptation post-CMI during physiological muscle shortening, which accompanies RLC, and MyBP-C phosphorylation change. These effects are evident at saturating calcium concentrations and maximal thin filament activation and therefore are at least partially independent of calcium sensitive regulation of the thin filament. Thus we identify a mechanism of sarcomeric compensation, which is mediated by changes in myosin function, created by sarcomeric protein phosphorylation change. These changes alter cross-bridge binding to actin and unbinding in a way that promotes increases in sarcomeric force, and power production. With these data we show the effect of CMI on myocardial contractile output during physiological shortening; providing a starting point to quantitatively assess the efficacy of interventions targeted to sarcomeric protein modification (23).

## GRANTS

The work presented herein was supported by Wellcome Trust Grants 091460/Z/10/Z and 092852/Z/10/Z and Biotechnology and Biological Sciences Research Council Grant BB/I019448/1.

## DISCLOSURES

No conflicts of interest, financial or otherwise, are declared by the author(s).

## AUTHOR CONTRIBUTIONS

C.N.T., V.C., K.T.M., T.G.W., and M.A.F. conception and design of research; C.N.T., M.B.S., V.C., A.V., I.T., and O.C. performed experiments; C.N.T., M.B.S., V.C., A.V., I.T., O.C., and T.G.W. analyzed data; C.N.T., M.B.S., V.C., A.V., I.T., O.C., T.G.W., and M.A.F. interpreted results of experiments; C.N.T., V.C., A.V., and I.T. prepared figures; C.N.T., M.B.S., V.C., A.R.L., S.B.M., P.K.L., K.T.M., T.G.W., and M.A.F. drafted manuscript; C.N.T., M.B.S., V.C., A.R.L., S.B.M., P.K.L., K.T.M., T.G.W., and M.A.F. edited and revised manuscript; C.N.T., T.G.W., and M.A.F. approved final version of manuscript.
